# Fingolimod treatment exacerbates tau phosphorylation and neurodegeneration in a mouse model of tauopathy with accumulated brain CD8^+^ T cells

**DOI:** 10.1093/braincomms/fcaf330

**Published:** 2025-09-25

**Authors:** Ryohei Uenishi, Rinna Kawata, Tatsuya Manabe, Toru Takeo, Masanori Hijioka, Takashi Saito

**Affiliations:** Department of Neurocognitive Science, Institute of Brain Science, Nagoya City University Graduate School of Medical Sciences, Mizuho-cho, Mizuho-ku, Nagoya, 467-8601, Japan; Department of Neurocognitive Science, Institute of Brain Science, Nagoya City University Graduate School of Medical Sciences, Mizuho-cho, Mizuho-ku, Nagoya, 467-8601, Japan; Department of Neurocognitive Science, Institute of Brain Science, Nagoya City University Graduate School of Medical Sciences, Mizuho-cho, Mizuho-ku, Nagoya, 467-8601, Japan; Division of Reproductive Engineering, Center for Animal Resources and Development, Kumamoto University, Kumamoto, 860-0811, Japan; Department of Neurocognitive Science, Institute of Brain Science, Nagoya City University Graduate School of Medical Sciences, Mizuho-cho, Mizuho-ku, Nagoya, 467-8601, Japan; Department of Neurocognitive Science, Institute of Brain Science, Nagoya City University Graduate School of Medical Sciences, Mizuho-cho, Mizuho-ku, Nagoya, 467-8601, Japan; Department of Neuroscience and Pathobiology, Research Institute of Environmental Medicine, Nagoya University, Nagoya, 464-8601, Japan

**Keywords:** Tauopathy, T cell, FTY720, Phosphorylated tau, Neurodegeneration

## Abstract

It is known that T cells play an important role in the progression of neurodegenerative disorders, including tauopathies. In this study, we used fingolimod (FTY720), an approved medication for the treatment of multiple sclerosis (MS), to manipulate T cell dynamics in P301S-Tau transgenic (Tau Tg) mice. FTY720 dramatically decreased the population of circulating T cells in blood. However, unexpectedly, we observed a marked increase in the number of CD8^+^ T cells in the hippocampus of FTY720-treated Tau Tg mice. This increase in CD8^+^ T cell number was significantly correlated with enhanced tau phosphorylation. Notably, FTY720-treated Tau Tg mice exhibited brain atrophy and neurodegeneration compared with controls. These findings indicate that CD8^+^ T cells in the brain contribute to the progression of tauopathies, and that brain CD8^+^ T cells may be a promising target for the treatment of tauopathies. This study provides new insights into the dynamics of brain T cells in neurodegenerative disorders. In addition, these results raise caution regarding FTY720 treatment in individuals predisposed to tauopathies, as it may promote neurodegeneration despite reducing peripheral T cells.

## Teaser

Uenishi *et al.* report that Fingolimod reduces peripheral T cells but increases brain CD8⁺ T cells in P301S-Tau transgenic mice, exacerbating tau pathology and neurodegeneration. These findings highlight CD8⁺ T cells as therapeutic targets for tauopathy and raise concerns about Fingolimod use in patients with multiple sclerosis predisposed to tauopathies.

## Graphical Abstract

**Graphical Abstract:**
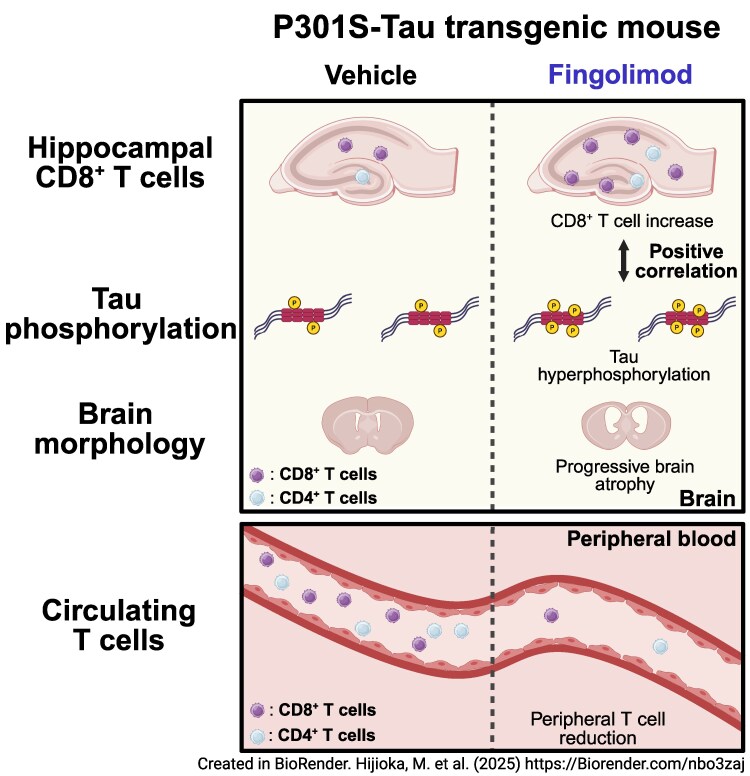

## Introduction

Tauopathies, including Alzheimer’s disease (AD), Pick disease (PiD), progressive supranuclear palsy (PSP) and corticobasal degeneration (CBD), are neurological and age-related neurodegenerative disorders. Such diseases are commonly characterized by abnormal accumulation of the microtubule-associated protein tau in the brain.^1^ In tauopathies, tau proteins become hyperphosphorylated, misfolded, and aggregate into abnormal structures, such as neurofibrillary tangles, leading to neuronal dysfunction and cell death. The correlation between phosphorylated tau accumulation and the progression of neurodegeneration suggests that removal of abnormal tau could be a promising therapeutic approach.^2^

Recent studies have reported the mobilization of immune cells, including T cells, in the brains of patients with tauopathies, highlighting the potential role of these cells in disease progression.^3-6^ It was reported that depletion of T cells using an anti-CD3 antibody suppressed tau phosphorylation and brain atrophy in a mouse model of tauopathy,^7^ suggesting the significant impact of T cells on pathogenesis. In this study, we used fingolimod (FTY720) as a pharmacological approach to modulate the number of peripheral circulating T cells. FTY720, which is clinically approved for the treatment of MS, works by reducing the level of circulating T cells by promoting their homing to lymph nodes.^8,9^ Herein, we investigated the effects of FTY720 on tau phosphorylation and brain atrophy in a mouse model of tauopathy.

## Materials and methods

### Animals and FTY720 treatment

All animal experiments were approved by the Institutional Animal Care and Use Committee of the Nagoya City University (approval number: 23-015) and conducted in accordance with relevant guidelines and regulations. In this research, the P301S-Tau transgenic (Line PS19) mice^10^ were used. These animals overexpress tau containing the mutations as those found in patients with frontotemporal dementia with Parkinsonism-17 (FTDP-17). The PS19 mice were back-crossed onto a C57BL/6J genetic background, with the resultant line called Tau Tg mice. Animals were housed in rooms with an 08:00 (lights on)—20:00 (lights off) light cycle and maintained at 22–25°C and 20–60% humidity, with ad libitum access to food and water. FTY720 (1 mg/kg) was administered intraperitoneally to male wild-type (WT) and Tau Tg mice daily for a month from 9 to 10 months of age. Prior to the injection, randomization of mice was performed and divided into the FTY720 or vehicle groups in a blinded manner.

### Sample preparation

Mice were deeply anesthetized by intraperitoneal injection of 0.75 mg/kg medetomidine, 4 mg/kg midazolam, and 5 mg/kg butorphanol and perfused transcardially with phosphate-buffered saline (PBS). After decapitation and removal of the whole brain, the right and left hemispheres were collected separately. The left hemisphere was soaked in 4% formaldehyde freshly prepared from paraformaldehyde at 4°C overnight. The hippocampus and cerebral cortex were isolated from the right hemisphere, snap frozen in liquid nitrogen, and stored at −80°C.

### Western blot

Brain tissue lysates were electrophoresed with 10% SDS polyacrylamide gels containing 2,2,2-trichloroethanol to detect total protein^11^ and transferred to PVDF membranes. Membranes were incubated with blocking buffer and then with primary antibodies overnight at 4°C. This was followed by incubation in horseradish peroxidase-conjugated secondary antibodies and ECL Prime western blotting detection reagent after which images of protein bands were obtained with a ChemiDoc Touch MP Imaging System. Stain-free total protein was fluorescence imaged for quantitative correction. The antibodies and equipment used are listed in Supplementary Tables 1 and 2.

### Immunohistochemistry

Fixed brain tissues were frozen and sectioned into 30 µm-thick slices. Three sections per mouse were mounted on glass slides and subjected to antigen retrieval using 10 mM citrate buffer (pH6.0) at 121°C for 5 min. The sections were then incubated with blocking buffer and subsequently with primary antibodies against CD8, CD31 and NeuN at 4°C overnight. Following this, the sections were incubated with secondary antibodies at room temperature, stained with 0.1% Sudan black, and washed in PBS.

Additionally, free-floating sections were incubated with blocking buffer and primary antibodies against CD4 and CD31 without antigen retrieval at 4°C overnight. After incubation with secondary antibody, these sections were mounted on glass slides. The antibodies and equipment used are listed in Supplementary Tables 1 and 2. Tile scan imaging of the hippocampus was performed using a confocal laser scanning microscope with a ×20 objective lens to identify CD4^+^ CD31^−^ and CD8^+^ CD31^−^ cells as brain parenchymal T cells. Images of Sudan black-stained and NeuN-immunostained sections were captured using a virtual slide scanner at ×40 magnification. Image analysis was carried out using QuPath software.

### Flow cytometry

Peripheral blood was collected from mice under deep anesthesia by cardiac puncture.^12^ The collected blood was mixed with erythrocyte lysis buffer and centrifuged. Precipitated cells were stained with fluorophore-conjugated antibodies and analyzed by FACS Aria III and FlowJo software. The antibodies and equipment used are listed in Supplementary Tables 1 and 2.

### Statistical analysis

All statistical analyses were performed using GraphPad Prism software with a significance threshold of *P* < 0.05. Data were presented as mean ± standard error of mean (S.E.M.). Normality was assessed using a Shapiro-Wilk test. Homogeneity of variances was evaluated by an *F* test (for two groups) or Brown-Forsythe test (for three or more groups). For the comparison of two groups, normally distributed data were analyzed using an unpaired two-tailed *t*-test, but otherwise using a Mann-Whitney U-test. For body temperature analysis, a two-way repeated measures analysis of variance (ANOVA) followed by a Šídák’s multiple comparisons test was performed. Meanwhile, when comparing among more than two groups, normally distributed data with equal variances were analyzed by a one-way ANOVA followed by a Tukey’s multiple comparisons test. Provided the assumption of homogeneity of variances was violated, Brown-Forsythe and Welch ANOVA tests were used followed by a Dunnett’s T3 multiple comparisons test. Alternatively, if the data did not follow the Gaussian distribution, a Kruskal-Wallis test was conducted with a Dunn’s multiple comparisons test.

## Results

### FTY720 reduces lymphocyte in peripheral blood

**Figure 1:**
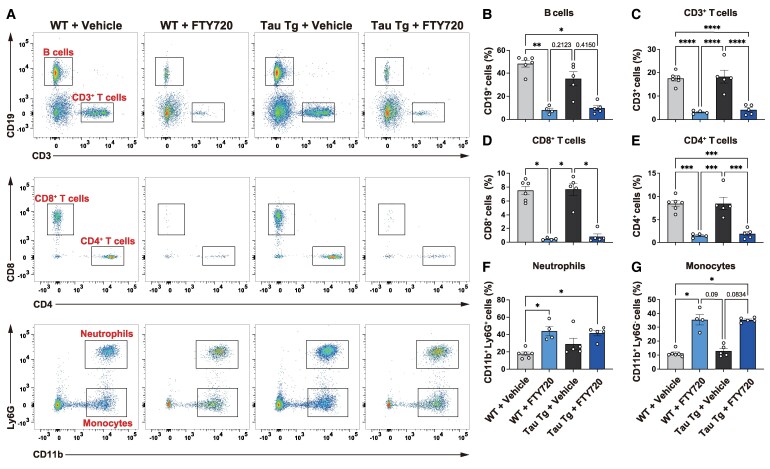
**Effect of FTY720 treatment on peripheral blood T cells.** (**A**) Peripheral blood analyzed by flow cytometry. (B–G) Quantification of cells in the singlet population: CD19^+^ B cells (**B**), CD3^+^ T cells (**C**), CD8^+^ T cells (**D**), CD4^+^ T cells (**E**), CD11b^+^ Ly6G^+^ neutrophils (**F**), and CD11b^+^ Ly6G^−^ monocytes (**G**). Data are mean ± S.E.M. Number of mice: WT + vehicle (*n* = 6), WT + FTY720 (*n* = 4), Tau Tg + vehicle (*n* = 5), Tau Tg + FTY720 (*n* = 5). Each datapoint represents an individual mouse sample. Statistical analysis was performed using the one-way ANOVA followed by the Tukey’s multiple comparisons test (C, E) and the Kruskal-Wallis test followed by the Dunn’s multiple comparisons test (B, D, F, G). (C) *F*(3, 16) = 26.09, *P* < 0.0001; (**E**) *F*(3, 16) = 23.17, *P* < 0.0001; (B) *H*(3) = 15.13, *P* = 0.0017; (**D**) *H*(3) = 14.24, *P* = 0.0026; (F) *H*(3) = 12.46, *P* = 0.0060; (**G**) *H*(3) = 14.26, *P* = 0.0026. *P < 0.05, **P < 0.01, ****P < 0.0001.

To confirm the effect of FTY720 on the circulating lymphocytes, we investigated immune cell populations in peripheral blood using flow cytometry (FCM). The main cell populations identified were CD19^+^ B cells, CD3^+^ T cells, CD4^+^ T cells, CD8^+^ T cells, CD11b^+^ Ly6G^+^ neutrophils, and CD11b^+^ Ly6G^−^ monocytes (Supplementary Fig. 1 and Fig. 1A). In both vehicle-treated WT and Tau Tg mice, the populations of all immune cells were at similar levels for both groups. FTY720 treatment significantly reduced the population of CD19^+^ B cells, CD3^+^ T cells, CD4^+^ T cells, and CD8^+^ T cells in WT mice. Concurrently, the proportions of CD11b^+^ Ly6G^+^ neutrophils and CD11b^+^ Ly6G^−^ monocytes were increased. These results were consistent with previous reports.^13,14^ In Tau Tg mice, FTY720 treatment also led to a reduction in lymphocytes, although the decrease in B cells did not reach statistical significance (Fig. 1B–G). These data suggest that FTY720 effectively reduces levels of peripheral lymphocytes, particularly T cells, in Tau Tg mice, while other immune cell populations remain unaffected.

### FTY720 increases CD8^+^ T cell numbers in the hippocampus of Tau Tg mice

**Figure 2:**
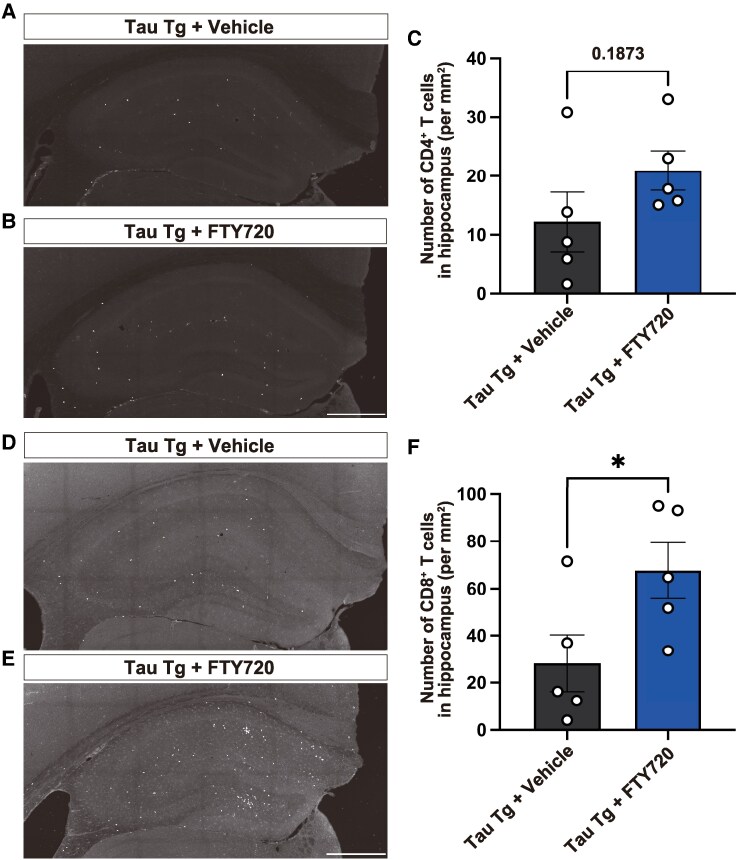
**Effect of FTY720 treatment on brain T cells in Tau Tg mice.** Representative images of CD4^+^ (A, B) or CD8^+^ (D, E) T cells in the hippocampus of vehicle and FTY720-treated Tau Tg mice (White). Scale bar = 500 µm. (C, F) Quantification of the number of CD4^+^ or CD8^+^ T cells in the hippocampus. Data are presented as mean ± S.E.M. Number of mice: Tau Tg + vehicle (*n* = 5), Tau Tg + FTY720 (*n* = 5). Each datapoint represents an individual mouse sample. Statistical analysis was performed using the unpaired two-tailed *t*-test. (**C**) *t*(8) = 1.442, *P* = 0.1873; (**F**) *t*(8) = 2.322, *P* = 0.0488. *P < 0.05.

We next examined the impact of FTY720 on the number of T cells in the hippocampus, as a higher number of T cells was observed in this region in comparison to other brain areas in Tau Tg mice. Unexpectedly, FTY720 treatment led to an increase in the number of both CD4^+^ and CD8^+^ T cells, with a statistically significant increase in CD8^+^ cells (Fig. 2A–F). T cells were not detected in the brains of WT mice, and similarly the changes seen in Tau Tg mice due to FTY720 treatment were also not observed in WT mice (data not shown). These findings indicate that the FTY720-related effect on CD8^+^ T cells occurs specifically in Tau Tg mice. Thus, FTY720 promotes an increase in the number of T cells in the hippocampus of Tau Tg mice independently of changes in peripheral blood.

### FTY720-induced T cell accumulation leads to increased tau phosphorylation

**Figure 3:**
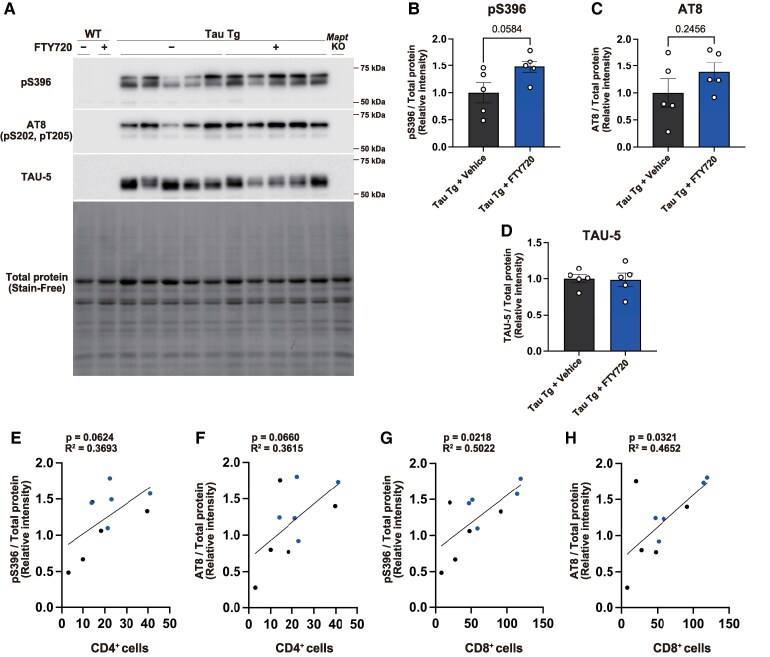
**Effect of FTY720 treatment on phosphorylated tau accumulation in Tau Tg mice.** (**A**) Immunoblot images of hippocampal tissue lysates of vehicle and FTY720-treated mice. Phosphorylated tau was detected with anti-phospho-tau (Ser396) antibody, and anti-phospho-tau (Ser202/Thr205) antibody [AT8]. Total tau was detected with anti-tau antibody [TAU-5]. Uncropped full-size membrane images of pS396, AT8, TAU-5, and total protein (Stain-Free) are shown in Supplementary Fig. 2 (A: pS396, B: AT8, C: TAU-5, D: Stain-Free). (B–D) Relative expression levels were quantified based on the band intensities of tau and normalized to total protein levels (Stain-Free). Data are presented as mean ± S.E.M., Number of mice: Tau Tg + vehicle (*n* = 5), Tau Tg + FTY720 (*n* = 5). Each datapoint represents an individual mouse sample. Statistical analysis was performed using an unpaired two-tailed *t*-test. (**B**) *t*(8) = 2.206, *P* = 0.0584; (**C**) *t*(8) = 1.253, *P* = 0.2456; (**D**) *t*(8) = 0.1483, *P* = 0.8858. (E–H) Black represents vehicle-treated Tau Tg mice, and Blue represents FTY720-treated Tau Tg mice, respectively. Number of mice: Tau Tg + vehicle (*n* = 5), Tau Tg + FTY720 (*n* = 5). Each datapoint represents an individual mouse sample. Statistical analyses were conducted simple linear regression. Linear regression analyses were performed to assess the relationship between the number of T cells (Fig. 2 C, F) and the relative expression levels of phosphorylated tau (Fig. 3B and C) in the hippocampus of Tau Tg mice. (**E**) *F*(1, 8) = 4.684, *P* = 0.0624, *R*² value = 0.3693; (**F**) *F*(1, 8) = 4.529, *P* = 0.0660, *R*² value = 0.3615; (**G**) *F*(1, 8) = 4.070, *P* = 0.0218, *R*² value = 0.5022; (**H**) *F*(1, 8) = 6.710, *P* = 0.0321, *R*² value = 0.4562.

Based on these observations, we investigated the effects of FTY720 treatment on the phosphorylation state of tau. Western blot analysis revealed that FTY720 treatment tended to promote tau phosphorylation in Tau Tg mice, but had no effect on total tau levels (Fig. 3A–D). Subsequently, we examined the correlation between the number of T cells in the brain and the relative expression levels of phosphorylated tau detected by western blots (Fig. 3B and C). Numbers of CD4^+^ and CD8^+^ T cells were both positively correlated with tau phosphorylation, with a significant correlation observed particularly in CD8^+^ T cells (Fig. 3E–H). These results suggest that the increase in T cells within the brains of Tau Tg mice may contribute to the enhancement of tau phosphorylation. Furthermore, because sphingosine-1-phosphate (S1P) stimulation induced a slight increase in body temperature in mice,^15^ FTY720, which inhibits the S1P signal pathway, exists the potential to cause hypothermia, namely a risk factor for elevating tau phosphorylation.^16^ To investigate this possibility, we monitored the body temperature of FTY720-treated WT mice. We found no change in body temperature when monitored every 4 h in 24 h (Supplementary Fig. 3).

### FTY720 causes atrophy and neurodegeneration in the hippocampus of Tau Tg mice

**Figure 4:**
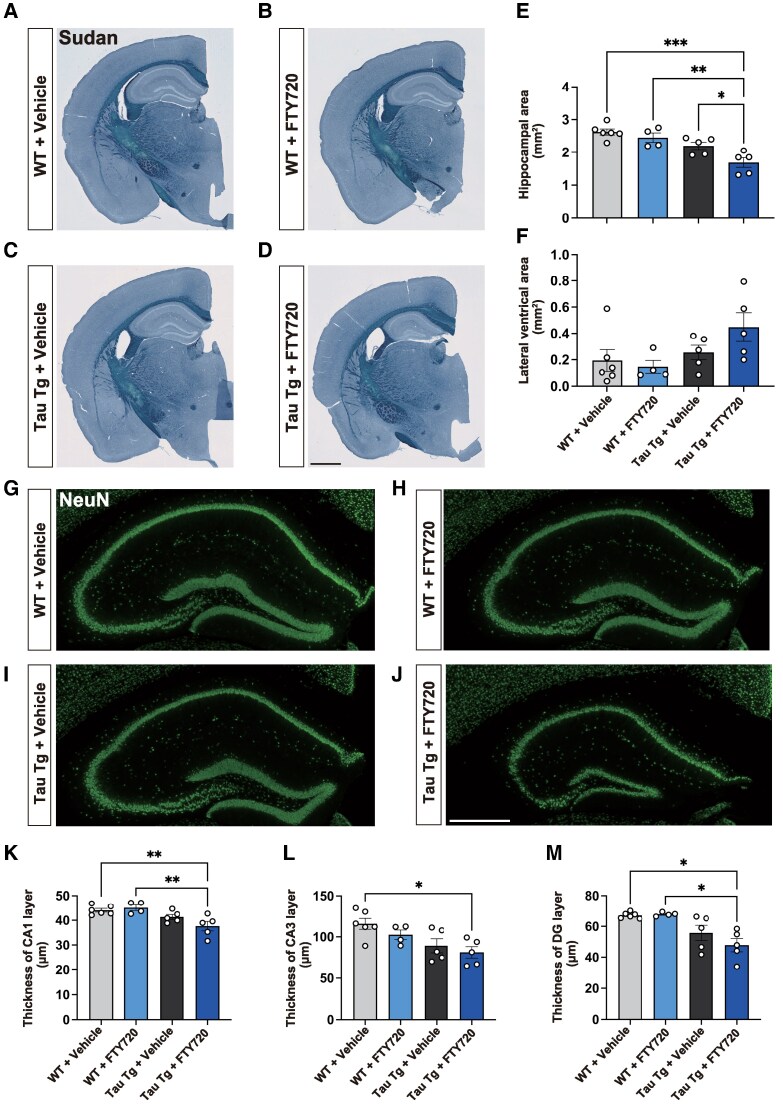
**Effect of FTY720 treatment on neurodegeneration in Tau Tg mice.** (**A–D**) Representative morphological images of brain coronal sections stained with Sudan Black. (**E**, **F**) Hippocampal area and lateral ventricle size were quantified from three sections per mouse and averaged. Scale bar = 1 mm. Data are presented as mean ± S.E.M., Number of mice: WT + vehicle (*n* = 6), WT + FTY720 (*n* = 4), Tau Tg + vehicle (*n* = 5), Tau Tg + FTY720 (*n* = 5). Each datapoint represents an individual mouse sample. Statistical analysis was performed using the one-way ANOVA followed by the Tukey’s multiple comparisons test (**E**) and Kruskal-Wallis test followed by the Dunn’s multiple comparisons test (**F**). (E) *F*(3, 16) = 11.75, *P* < 0.0003; (**F**) *H*(3) = 5.972, *P* = 0.1130. *P < 0.05, **P < 0.01, ***P < 0.001. (G–J) Representative images of NeuN-immunopositive cells in the hippocampus (Green). Scale bar = 500 µm. (K–M) Quantification of the thickness of CA1 (**K**), CA3 (**L**), DG (**M**) layers. Number of mice: WT + vehicle (*n* = 6), WT + FTY720 (*n* = 4), Tau Tg + vehicle (*n* = 5), Tau Tg + FTY720 (*n* = 5). Each datapoint represents an individual mouse sample. Statistical analysis was performed using the one-way ANOVA followed by the Tukey’s multiple comparisons test (K, L) and Brown-Forsythe and Welch ANOVA tests to account for unequal variances across groups followed by the Dunnett’s T3 multiple comparisons test (**M**). (**K**) *F*(3, 16) = 6.787, *P* < 0.0037; (**L**) *F*(3, 16) = 4.861, *P* < 0.0137; (**M**) Brown-Forsythe ANOVA: *F*(3, 8.238) = 8.352, *P* = 0.0071, Welch's ANOVA: *W*(3, 8.069) = 7.618, *P* = 0.0097. *P < 0.05, **P < 0.01.

Lastly, we confirmed the presence of regional brain atrophy and neurodegeneration in FTY720-treated Tau Tg mice. In comparison with the untreated group, Tau Tg mice showed mild atrophy of the hippocampus compared with WT mice, but the difference was not statistically significant. However, FTY720 treatment led to hippocampal shrinkage and lateral ventricle enlargement in Tau Tg mice as evidenced by a significantly reduced hippocampal volume. (Figure 4A–F). Furthermore, we quantified the thickness of NeuN-positive cell layers in the CA1, CA3, and dentate gyrus (DG). The reduction in thickness of each region was observed exclusively in FTY720-treated Tau Tg mice (Fig. 4G–M). These findings suggest that FTY720 accelerates the progression of hippocampal atrophy and neurodegeneration in Tau Tg mice.

## Discussion

Based on the results of many previous studies, strategies such as removing insoluble tau through antibody therapy and the modification of the molecular properties of tau, including post-translational modifications, have been considered promising for the treatment of tauopathy.^17,18^ However, despite many potential agents reaching advanced clinical trial stages, most approaches have not been successful. Against this background, the identification of new therapeutic targets has become an important issue. Recent research has highlighted the role of T cell-mediated microglial responses in disease progression within Tau Tg mice, demonstrating the importance of understanding brain T cell dynamics in the context of neurodegenerative diseases.^19,20^

In this study, we administered the clinically approved drug FTY720, which can manipulate the dynamics of T cells, to a tauopathy mouse model to investigate the role of T cells in the pathogenesis of tauopathy. Unexpectedly, treatment with FTY720 reduced lymphocyte levels in the peripheral blood but led to an increase of T cells—particularly CD8^+^ T cells—in the brain. A significant correlation was observed between the number of CD8^+^ T cells in the hippocampus and levels of phosphorylated tau, suggesting that these T cells may facilitate the progression of tau pathology. FTY720 administration was also associated with hippocampal atrophy and neurodegeneration, as evidenced by a reduction in NeuN^+^ cell layers, highlighting the pivotal role of CD8^+^ T cells in the progression of tauopathies. Of particular importance, a case report has documented the occurrence of a tumefactive demyelinating lesion in an MS patient treated with FTY720. In this case, an increase in CD8^+^ effector T cells (CD45RO⁻CCR7⁻) in the cerebrospinal fluid (CSF) was also observed.^21^ Together with our findings, these suggest that treatment with FTY720 may exacerbate MS symptoms if there is a latent predisposition to, for example, tauopathy, which is hidden by MS symptoms. In this way, it suggests that clinical treatment with FTY720 may promote the accumulation of CD8^+^ T cells in the brain and accelerate neurodegeneration, supporting our hypothesis that CD8^+^ T cells play an important role in neurodegeneration.

The detailed molecular and cellular mechanisms underlying the relationship between the increase in brain T cells and the phosphorylated tau accumulation in FTY720-treated Tau Tg mice remain unclear. As a possible explanation for the increase in T cell number in the brain, the compromised neurovascular unit is seen in conditions like AD and Parkinson’s disease, where activated glial cells, including astrocytes and microglia, produce chemokines that attract peripheral T cells into the brain.^22^ However, our findings suggested that no correlation exists between peripheral and brain T cell levels following FTY720 treatment, indicating that this mechanism does not apply.

Another possible route for T cell infiltration is through the CSF and meninges. Some single-cell RNA sequencing (scRNA-seq) studies have identified immune cells in the CSF of AD patients and the meninges of tauopathy models.^7,23,24^ Nevertheless, we could not analyze the migration of T cells from the CSF or meninges in FTY720-treated Tau Tg mice, leaving the exact migration pathway uncertain.

A third hypothesis is that memory T cells undergo clonal expansion within the brain upon antigen presentation. Tissue-resident memory T cells (TRM) are particularly implicated in this process, as they remain in local tissues to provide rapid immune responses to invading pathogens.^25^ Evidence of CD8^+^ TRM in human and mouse brains,^26,27^ along with an increase in antigen-presenting microglia expressing MHC class II genes under disease conditions,^28^ support this theory. Additionally, we detected a small number of proliferating T cells expressing Ki67 in the brains of FTY720-treated Tau Tg mice, hinting at possible clonal expansion.^29^ In addition, no changes in T cells were observed in the brains of WT mice treated with FTY720 (data not shown). Future investigations will need to confirm this through T cell receptor repertoire analysis and the identification of specific antigen-presenting cells in the brain.

Alternatively, tau phosphorylation is tightly regulated by body temperature change whereby hypothermia results in hyperphosphorylation.^16^ S1P—an agonist that binds to the same receptor as FTY720—can raise the body temperature if injected over an intracerebroventricular route.^15^ It was reasonable to speculate that FTY720 could lead to hypothermia by inhibiting the S1P signal pathway and consequently elevate tau phosphorylation in the brain. However, when the rectal temperature was recorded after the FTY720 injection every 4 h in 24 h, the body temperature was unaffected. This implies that the contributions of hypothermia in this study should be negligible.

Our conclusion is also limited by the use of a single animal model of tauopathy. Tau Tg mice overexpress the P301S mutant tau that causes FTDP-17 in humans but partially shares the tau pathology with patients affected by PSP, CBD, and AD. Recent cryo-electron microscopy studies also confirmed the presence of structurally distinct tau filaments in this model.^30,31^ Since the presence of T cells in the brains was previously documented in patients with tauopathy,^6^ our observations are likely transferrable to these patients but will need further validation using alternative approaches. Inoculation of patient-derived tau aggregates into the mouse brain is a case in point because this results in the propagation of hyperphosphorylated tau.^32^ Furthermore, our model does not develop Aβ pathology in the brain. It would be intriguing to treat FTY720 with mouse models of Aβ amyloidosis to investigate whether it also causes tau phosphorylation in harboring a predisposition of Aβ pathology.

In summary, FTY720 treatment led to a reduction in peripheral T cells but increased brain T cells in Tau Tg mice, thereby aggravating tau pathology. These findings suggest that targeting brain T cells could be a promising therapeutic strategy to combat tauopathies. Understanding the molecular and cellular mechanisms that promote an increase in T cells in the brain is crucial for developing novel treatments for neurodegenerative diseases.

## Supplementary Material

fcaf330_Supplementary_Data

## Data Availability

Raw data obtained in this study are available upon request. As no data requiring source code or similar resources were generated, such data sharing is not applicable to this work.
