# Supplementary material for: Fingolimod treatment exacerbates tau phosphorylation and neurodegeneration in a mouse model of tauopathy with accumulated brain CD8+ T cells
Source: Brain Commun. 2025 Sep 25;7(5):fcaf330. doi: 10.1093/braincomms/fcaf330 (PMC12459988; doi:10.1093/braincomms/fcaf330)
Supplement: fcaf330_Supplementary_Data [file fcaf330_supplementary_data.pdf]

# Fingolimod treatment exacerbates tau phosphorylation and neurodegeneration in a mouse model of tauopathy with accumulated brain $CD8^+$ T cells


## Author and affiliations:

Ryohei Uenishi[1], Rinna Kawata[1], Tatsuya Manabe[1], Toru Takeo[2], Masanori Hijioka[1*], Takashi Saito [1,3*]

1. Department of Neurocognitive Science, Institute of Brain Science, Nagoya City University Graduate School of Medical Sciences, Nagoya, Japan
2. Division of Reproductive Engineering, Center for Animal Resources and Development, Kumamoto University, Kumamoto, Japan
3. Department of Neuroscience and Pathobiology, Research Institute of Environmental Medicine, Nagoya University, Nagoya, Japan

## *Correspondence to:

Drs. Masanori Hijioka and Takashi Saito


## Contents:


Supplementary Figures 1–3

Supplementary Tables 1, 2

## Supplementary Figure 1

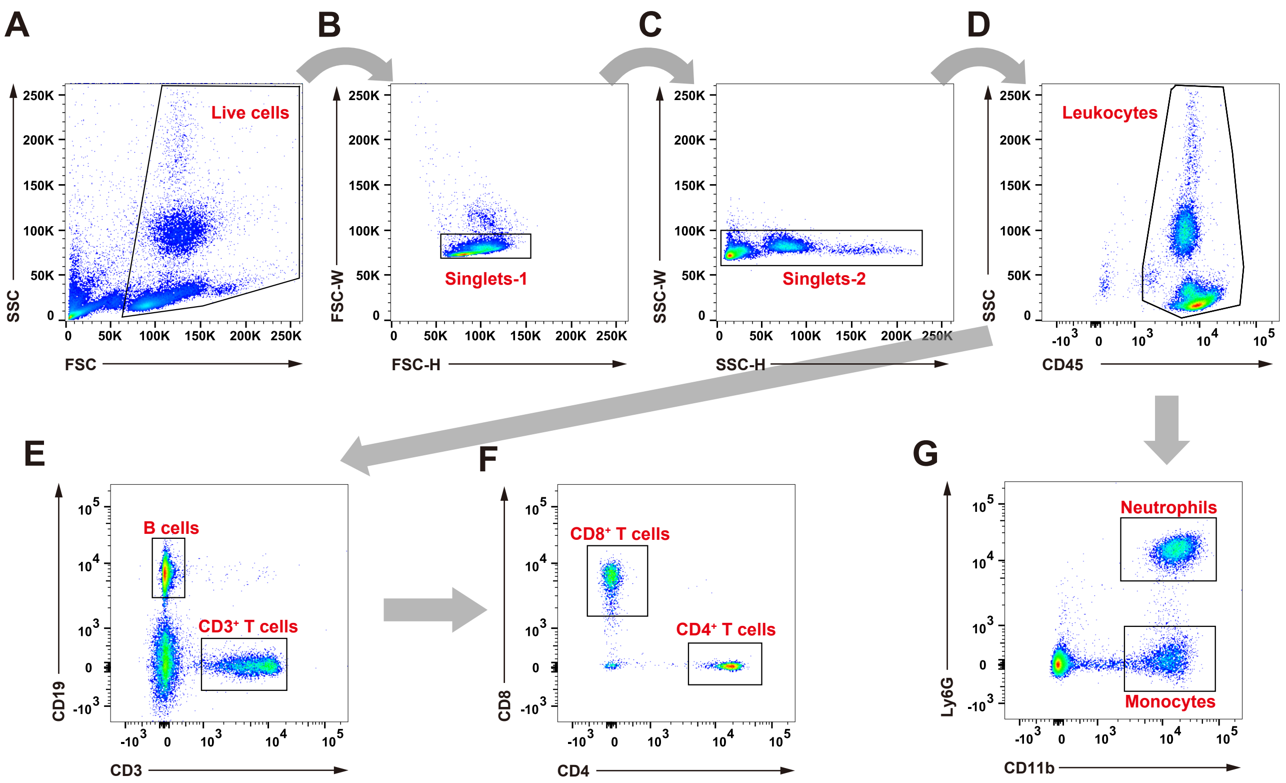


Supplementary Figure 1. Gating strategy for flow cytometry analysis of leukocytes in hemolyzed peripheral blood.

Data from one vehicle-treated WT mouse were used as a representative flow cytometry result. Sequential gating was performed on live cells (A), singlets (B, C), and CD45$^{+}$ cells (D). CD19$^{+}$ B cells, CD3$^{+}$ T cells (E), and myeloid cells (G) were subclustered from CD45$^{+}$ cells (D). CD4$^{+}$ and CD8$^{+}$ T cells (F) were identified from the CD3$^{+}$ cell population.

# Supplementary Figure 2

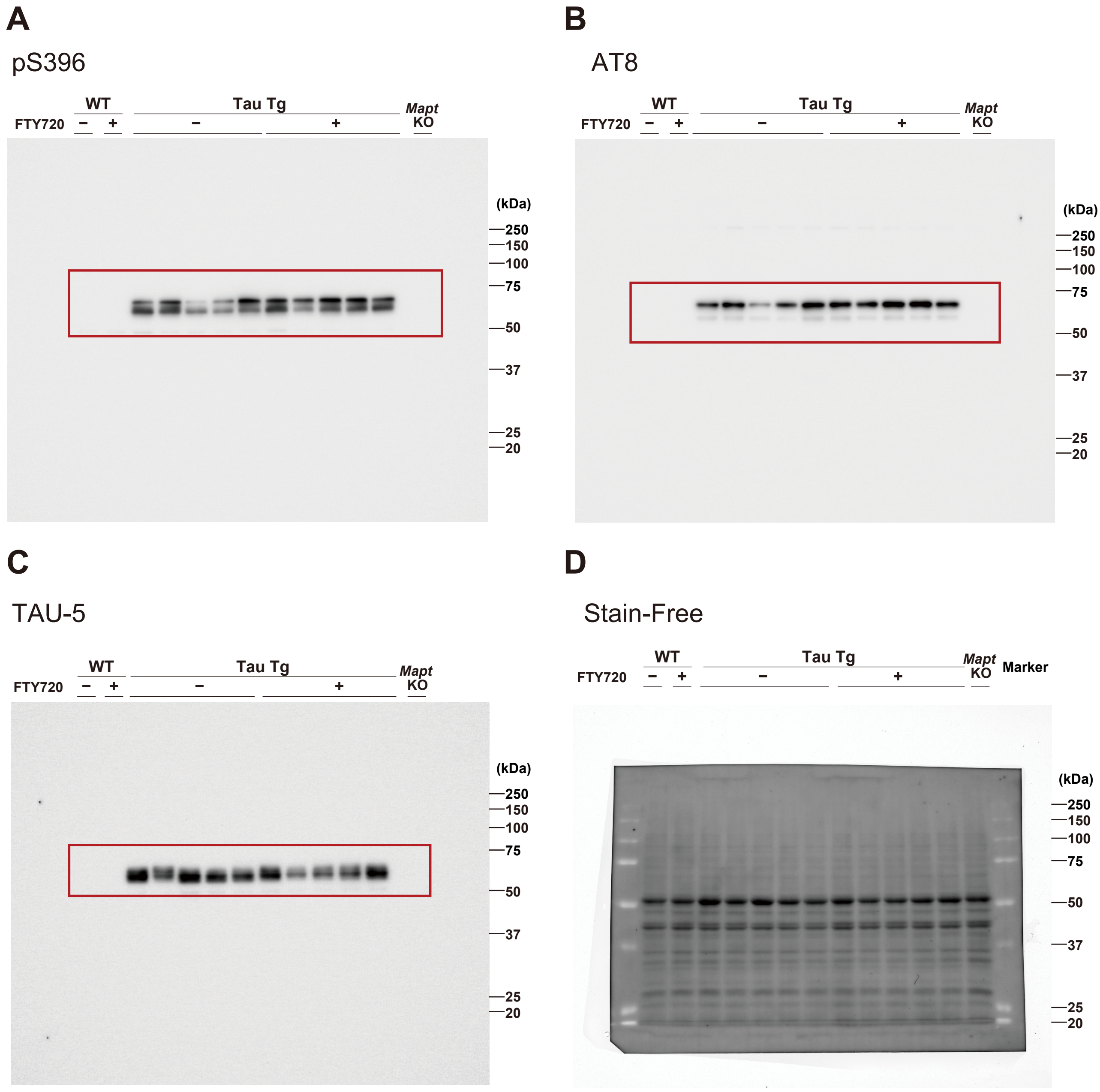


Supplementary Figure 2. Uncropped full-size membrane images of WB from Figure 3.

(A–C) Protein bands were detected using specific primary antibodies to pSer396, AT8, and TAU-5, respectively. (D) Total protein was visualized using the Stain-Free method.

**Supplementary Figure 3**

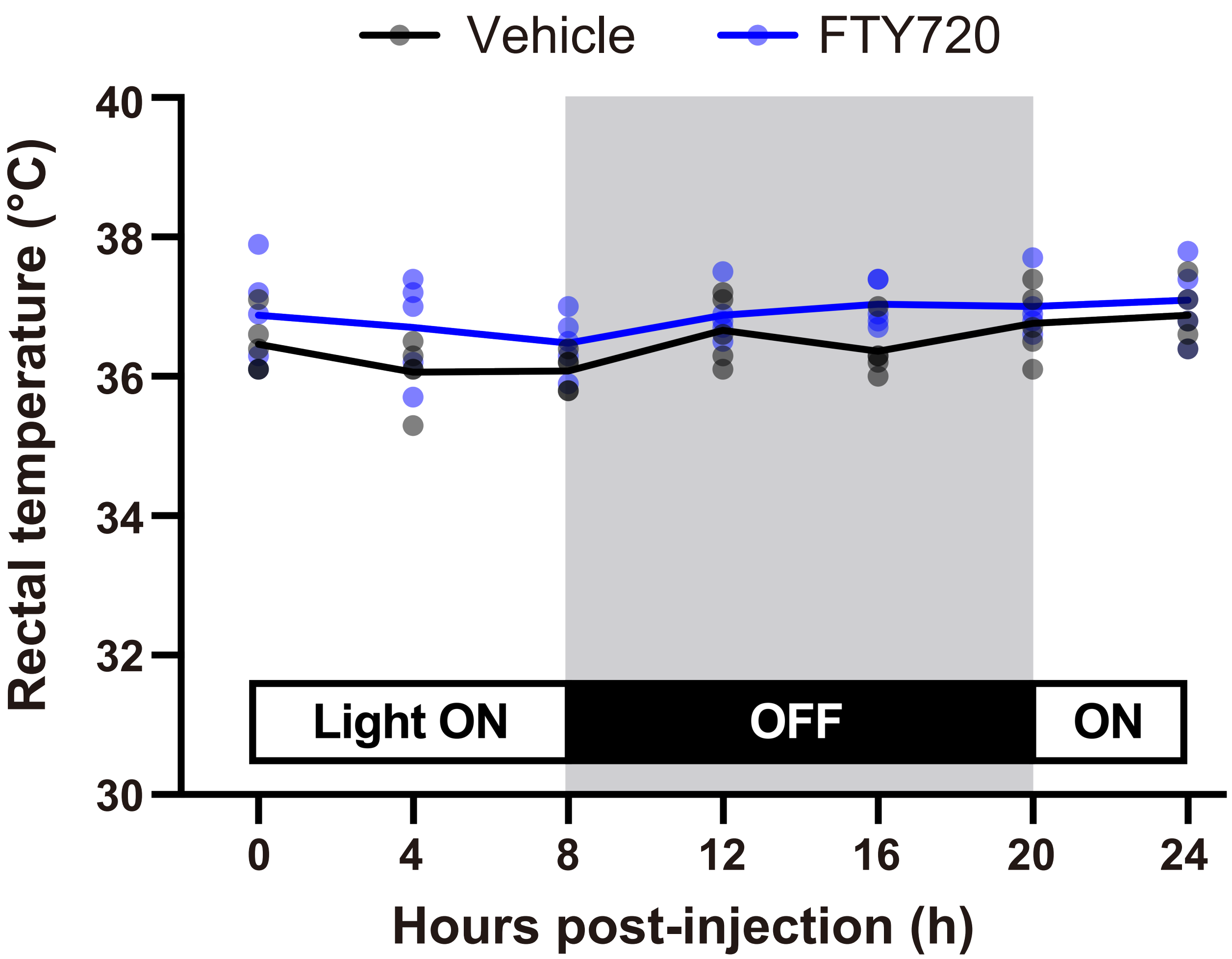


Supplementary Figure 3. Body temperature following FTY720 administration.

Rectal temperature was measured at 4-hour intervals for over 24 hours after FTY720 administration. Black line: vehicle-treated mice; blue line: FTY720-treated mice. Number of mice: WT + vehicle (n = 5), WT + FTY720 (n = 5). Each datapoint represents an individual mouse sample. Statistical analysis was performed using the two-way repeated measures ANOVA followed by the Šídák's multiple comparisons test. Time × Treatment: $F(6, 48) = 0.592$, $p = 0.7349$; Time: $F(6, 48) = 4.11$, $p = 0.0021$; Treatment: $F(1, 8) = 4.39$, $p = 0.0694$; Subject: $F(8, 48) = 4.05$, $p = 0.0010$.

## Supplementary Table 1

| Antibodies | Source | Dilution | Identifier |
|---|---|---|---|
| **for IHC** | | | |
| anti-mouse CD4 IgG [RM4-5] | BioLegend | 1: 500 | Cat# 100506, RRID:AB_312709 |
| anti-mouse CD8a IgG [4SM16] | Thermo Fisher Scientific | 1: 500 | Cat# 14-0195-80, RRID:AB_2637158 |
| anti-mouse/rat CD31/PECAM1 IgG | R&D Systems | 1: 500 | Cat# AF3628, RRID:AB_2161028 |
| anti-rat IgG (H+L) Highly Cross-Adsorbed Secondary Antibody, Alexa Fluor™ 488 | Thermo Fisher Scientific | 1: 1,000 | Cat# A-21208, RRID:AB_2535794 |
| anti-goat IgG (H+L) Cross-Adsorbed Secondary Antibody, Alexa Fluor™ 568 | Thermo Fisher Scientific | 1: 1,000 | Cat# A-11057, RRID:AB_2534104 |
| **for WB** | | | |
| anti-human Phospho-Tau (Ser202, Thr205) IgG [AT8] | FUJIREBIO | 1: 1,000 | Cat# 90206, RRID: AB_223648 |
| anti-human/mouse/rat phospho-Tau (Ser396) IgG | Thermo Fisher Scientific | 1: 5,000 | Cat# 44-752G, RRID:AB_2533745 |
| anti-human/mouse/rat Tau IgG [TAU-5] | Abcam | 1: 1,000 | Cat# ab80579, RRID:AB_1603723 |
| anti-mouse IgG (H+L), Horseradish Peroxidase | Jackson ImmunoResearch | 1: 20,000 | Cat# 715-035-150, RRID:AB_2340770 |
| anti-rabbit IgG (H+L), Horseradish Peroxidase | Jackson ImmunoResearch | 1: 20,000 | Cat# 711-035-152, RRID:AB_10015282 |
| **for FCM** | | | |
| anti-mouse CD45 IgG [30-F11], FITC | BioLegend | 1: 400 | Cat# 11-0451-82, RRID:AB_465050 |
| anti-mouse/human CD11b IgG [M1/70], PE/Cyanine7 | BioLegend | 1: 800 | Cat# 101216, RRID:AB_312799 |
| anti-mouse CD19 IgG [6D5], Brilliant Violet 785™ | BioLegend | 1: 100 | Cat# 115543, RRID:AB_11218994 |
| anti-human/mouse CD3e IgG [145-2C11], APC | Thermo Fisher Scientific | 1: 400 | Cat# 17-0031-82, RRID:AB_469315 |
| anti-mouse CD8a IgG [53-6.7], Brilliant Violet 510™ | Becton Dickinson | 1: 100 | Cat# 563068, RRID:AB_2687548 |
| anti-human/mouse CD4 IgG [RM4-5], PE | Thermo Fisher Scientific | 1: 400 | Cat# 12-0042-82, RRID:AB_465510 |
| anti-mouse Ly6G IgG [1A8], APC/Cyanine7 | BioLegend | 1: 100 | Cat# 127623, RRID:AB_10645331 |

Supplementary Table 1. List of antibodies used for IHC, WB, and FCM.

## Supplementary Table 2

| Reagent or Resource | Source | Identifier |
|---|---|---|
| **Mouse** | | |
| C57BL/6J | Jackson Laboratory | Cat# 000664, RRID:IMSR_JAX:000664 |
| B6.Cg-Tg(Prnp-MAPT*P301S)PS19Vle/J | Dr. Virginia M Y Lee | Cat# 024841, RRID:IMSR_JAX:024841 |
| **Compound** | | |
| Fingolimod | Cayman Chemical | Cat# 10006292 |
| **Equipment** | | |
| Multi-beads shocker | Yasui Kikai | Cat# MB3000 |
| ChemiDoc™ Touch MP imaging system | Bio-Rad Laboratories | Cat# 17001402JA, RRID:SCR_021693 |
| FV3000 confocal laser scanning microscope | Evident Scientific | Cat# 7M88501, RRID:SCR_017015 |
| Virtual slide scanner NanoZoomer S60 | Hamamatsu Photonics | C13210-01, RRID:SCR_023762 |
| FACSAria III Cell Sorter | Becton Dickinson | Cat# 648282, RRID:SCR_016695 |
| FlowJo | Becton Dickinson | Cat# 663335, RRID:SCR_008520 |
| GraphPad Prism (version 10.4.0) | GraphPad Software | RRID:SCR_002798 |

Supplementary Table 2. List of materials other than antibodies.
